# Serum syndecan-1 correlates with coronary artery calcification severity and intradialytic hypotension in elderly hemodialysis patients

**DOI:** 10.3389/fcvm.2026.1842977

**Published:** 2026-06-11

**Authors:** Dian Zhu, Yiyuan Zhang, Yu Song, Yu Xi, Mingzhe Zhu, Hailun Li, Donghui Zheng, Xue Zhao

**Affiliations:** 1 Department of Nephrology, The Affiliated Huai'an Hospital of Xuzhou Medical University and Huai'an Second People's Hospital, Huai'an, China; 2 Department of Nephrology, The Affiliated Huaian No. 1 People's Hospital of Nanjing Medical University, Huai'an, China; 3 Special Needs Ward, The Affiliated Huai'an Hospital of Xuzhou Medical University and Huai'an Second People's Hospital, Huai'an, China

**Keywords:** coronary artery calcification, endothelial glycocalyx, hemodialysis, intradialytic hypotension, syndecan-1

## Abstract

**Background and objectives:**

Cardiovascular disease is the leading mortality cause in elderly maintenance hemodialysis (MHD) patients, driven by coronary artery calcification (CAC). Elevated serum syndecan-1 (SDC1), a biomarker of endothelial glycocalyx degradation, indicates vascular dysfunction. This study explored the cross-sectional associations between serum SDC1, CAC severity, and intradialytic hypotension (IDH) in this population.

**Materials and methods:**

This cross-sectional study enrolled 88 elderly MHD patients and 30 healthy controls. Serum SDC1 was quantified, and CAC severity was assessed via computed tomography coronary artery calcium scores (CACS). IDH episodes were retrospectively reviewed from 3-month standardized dialysis records. Associations were analyzed using Spearman's correlation and multivariable ordinal logistic regression with a clinically prespecified sequential adjustment strategy. The discriminative stability of SDC1 was internally validated using the bootstrap resampling method.

**Results:**

Serum SDC1 was significantly higher in MHD patients than controls (*P* < 0.001). SDC1 strongly correlated with CACS (*r* = 0.75, *P* < 0.001), increasing progressively across CAC severity strata. After adjusting for demographics, clinical history, and mineral metabolism markers, elevated SDC1 was independently associated with greater CAC severity (Adjusted OR = 1.148, 95% CI 1.096–1.202, *P* < 0.001). Exploratory ROC analysis yielded a robust bootstrap-validated AUC of 0.920 (95% CI 0.867–0.974). An exploratory SDC1 threshold of 33.5 pg/mL identified a phenotype with higher CAC prevalence (86.9% vs. 7.4%, *P* < 0.001) and more frequent IDH episodes (*P* < 0.001).

**Conclusions:**

Elevated serum SDC1 is independently associated with CAC severity and IDH incidence in elderly MHD patients. These findings suggest that SDC1, reflecting endothelial glycocalyx degradation, may serve as a potential indicator of vascular and hemodynamic instability. However, its exploratory role in risk assessment requires further prospective validation in independent cohorts before clinical application.

## 1 Introduction

Cardiovascular disease (CVD) remains the predominant cause of mortality among patients with end-stage kidney disease (ESKD), particularly in the elderly population undergoing maintenance hemodialysis (MHD), where the risk is elevated up to tenfold compared to the general population (1, 2). A key pathological driver of this excessive risk is coronary artery calcification (CAC), which is widely recognized as an independent predictor of cardiovascular events and all-cause mortality (3, 4). Within the Chinese dialysis population, the prevalence and severity of CAC increase markedly with prolonged dialysis vintage, adversely impacting clinical outcomes and quality of life.

While traditional disorders of mineral and bone metabolism (CKD-MBD) partially explain this phenomenon, endothelial dysfunction has been increasingly implicated as a crucial early contributor to the pathogenesis of vascular calcification (5–7). The endothelial glycocalyx, a fragile layer lining the vascular lumen, plays a crucial role in maintaining vascular homeostasis by regulating permeability, inhibiting inflammation, and preventing thrombosis (8–10). Its degradation is one of the earliest detectable markers of endothelial injury. Syndecan-1 (SDC1), a core proteoglycan component shed upon glycocalyx disruption, has emerged as a sensitive circulating biomarker reflecting this specific damage (11).

Although elevated serum SDC1 levels have been associated with atherosclerosis and adverse cardiovascular outcomes in broader patient cohorts, the precise relationship between glycocalyx damage—quantified by SDC1—and the severity of CAC in elderly MHD patients remains inadequately defined (12, 13). This specific population endures relentless endothelial insults not only from circulating uremic toxins but also from the non-physiological shear stress of the hemodialysis procedure itself. Furthermore, while chronic endothelial damage is closely linked to structural calcification, the acute loss of glycocalyx integrity may simultaneously compromise endothelium-dependent vasomotor regulation. This acute dysfunction could link SDC1 shedding to broader clinical manifestations of hemodynamic instability, such as intradialytic hypotension (IDH), a relationship that has not yet been systematically investigated.

Therefore, this study aimed to explore the cross-sectional association between serum SDC1 levels and CAC severity in elderly MHD patients and to descriptively evaluate its potential discriminative value. A secondary objective was to explore the association of SDC1 with clinical markers of hemodynamic instability, specifically IDH.We hypothesize that SDC1 may serve as a preliminary biomarker reflecting endothelial glycocalyx degradation, which may be associated with both chronic vascular pathology and acute clinical vulnerability in this patient population. This study is intended to be exploratory, aiming to generate hypotheses for future prospective validation.

## 2 Materials and methods

### 2.1 Study design and participants

**Figure 1:**
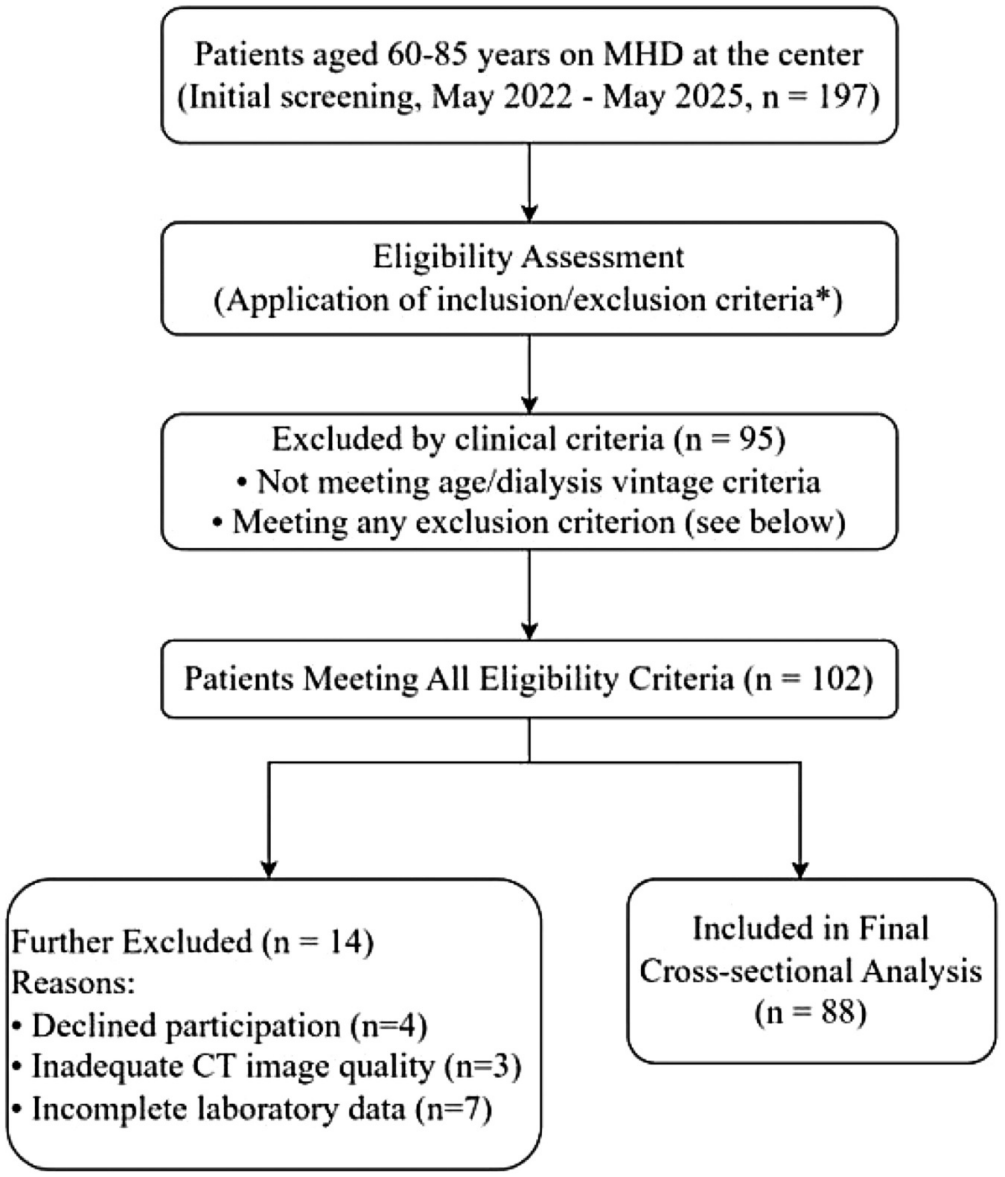
Flowchart of patient screening, eligibility assessment, and enrollment. A total of 197 elderly patients on maintenance hemodialysis (MHD) were initially screened between May 2022 and May 2025. Following the systematic application of inclusion and exclusion criteria (e.g., history of coronary intervention, structural heart disease, or active infection), 88 patients were enrolled in the final cross-sectional analysis. Additionally, 30 age-matched healthy volunteers were recruited as a control group for biomarker comparison. The sample size was determined based on a predefined power analysis (*r* *=* *0.6,* power = 80%).

This single-center, exploratory,cross-sectional study consecutively enrolled patients aged 60–85 years undergoing maintenance hemodialysis (MHD) at the Blood Purification Center of Huai'an Hospital, affiliated with Xuzhou Medical University, between May 2022 and May 2025. The patient selection process is outlined in Figure 1. Inclusion criteria were: (1) age 60–85 years; (2) MHD treatment for ≥ 3 months; and (3) clinical referral for coronary multidetector computed tomography (MDCT) due to atypical chest pain. Key exclusion criteria included a history of coronary intervention or myocardial infarction, significant arrhythmias (e.g., atrial fibrillation), prior parathyroidectomy, structural heart disease, active infection, or malignancy.

The sample size was estimated based on an expected correlation coefficient (*r* = 0.6) between serum SDC1 and CACS. With a two-sided *α* of 0.05 and a power of 80%, a minimum of 76 patients was required; 88 patients were ultimately included to ensure robust statistical power. Additionally, to establish a physiological baseline for serum SDC1, 30 age-matched healthy controls were enrolled using a two-step approach. First, individuals with normal renal function who had recently undergone routine Coronary Computed Tomography Angiography (CCTA) during comprehensive health check-ups were retrospectively screened from our hospital's Physical Examination Center. Only subjects with a confirmed zero coronary artery calcium score (CACS = 0) were selected. Subsequently, these identified individuals were prospectively enrolled, and fasting blood samples were collected to measure their baseline SDC1 levels.

### 2.2 Ethical approval and data availability

The study protocol was approved by the Ethics Committee of the Affiliated Huai'an Hospital of Xuzhou Medical University (No.: HEYLL202204). All procedures adhered to the Declaration of Helsinki, and written informed consent was obtained from all participants. De-identified datasets are available from the corresponding author upon reasonable request. Generative artificial intelligence was not utilized in any phase of this research.

### 2.3 Data collection and laboratory measurements

Fasting venous blood samples were collected from MHD patients on a mid-week non-dialysis day after an 8-hour fast. Routine biochemical parameters were analyzed using a Toshiba automated analyzer. Serum SDC1 concentration was quantified via a commercial ELISA kit (Shanghai Kanglang Biotechnology Co., Ltd.) following the manufacturer's protocol. The intra-assay and inter-assay coefficients of variation were consistently below 10%, ensuring measurement precision.

### 2.4 Assessment of coronary artery calcification

Coronary Artery Calcification (CAC) was quantitatively assessed via the Coronary Artery Calcium Score (CACS). Imaging was performed using a 64-slice spiral CT scanner with a prospective ECG-gated technique. CACS was calculated using the Agatston method (threshold ≥ 130 HU, area ≥ 1 *mm*²). All scans were independently adjudicated by two blinded radiologists, with disagreements resolved by consensus. Participants were categorized as CAC-negative (CACS = 0) or CAC-positive (CACS > 0), with the latter further stratified into mild (1–100), moderate (101–400), and severe (> 400) groups.

### 2.5 Definition and assessment of intradialytic hypotension

Intradialytic hypotension (IDH) was defined as a symptomatic systolic blood pressure (SBP) drop ≥ 20 mmHg or a nadir SBP < 90 mmHg necessitating nursing intervention. The monitoring protocol is summarized in Supplementary Figure S1. To minimize retrospective bias, all dialysis records were retrieved from a standardized electronic recording system. These records were consistently maintained by trained nursing staff using unified documentation requirements for IDH episodes and interventions. We documented IDH frequency and management strategies (e.g., saline infusion, ultrafiltration adjustment) over the 3 months preceding enrollment.

### 2.6 Statistical analysis

Analyses were performed using SPSS (v26.0) and R (v4.3.1). Continuous variables are presented as mean ± standard deviation or median [interquartile range] based on normality. Group comparisons utilized *t*-tests, ANOVA, or non-parametric tests as appropriate.Correlations were assessed using Spearman's rank correlation coefficients depending on data distribution, and visualized via a heatmap (Supplementary Figure S2). To evaluate the independent association between SDC1 and CAC severity while preventing model overfitting, multivariable ordinal logistic regression was employed using a clinically prespecified sequential adjustment strategy. Models were adjusted for demographics (Model 1), clinical history including diabetic nephropathy (Model 2), and core CKD-MBD markers (Model 3). Collinearity among covariates was ruled out by calculating the Variance Inflation Factor (VIF). Restricted cubic spline (RCS) analysis visualized the non-linear relationship between SDC1 and CAC risk.Exploratory ROC curve analysis evaluated the discriminative ability of SDC1. To rigorously validate the model's stability and the optimal cut-off (determined by the maximum Youden's index), internal validation was performed using the bootstrap resampling method with 1,000 iterations via the pROC package in R.A two-tailed *P* < 0.05 was considered statistically significant.

## 3 Results

### 3.1 Demographic and clinical features of participants

A total of 197 elderly MHD patients were initially screened. Following the application of eligibility criteria and exclusions (as outlined in Figure 1), the final cross-sectional analysis comprised 88 patients. The cohort had a median age of 69 years (IQR 62.8–78.0), with 61.4% (54 patients) being male. The median dialysis vintage was 26.2 months (IQR 20.3–39.6). Diabetic nephropathy (47.7%) and chronic nephritis (19.3%) were the most common primary causes of ESKD.

Patients were dichotomized into a CAC-positive group (*n* = 55, 62.5%) and a CAC-negative group (*n* = 33, 37.5%) based on their CACS. Compared to 30 age-matched healthy controls(retrospectively screened to confirm a zero CAC score), MHD patients exhibited significantly higher serum SDC1 levels [71.73 [45.80, 79.30] vs. 25.24 [22.05, 33.46] pg/mL, *P* < 0.001] and a markedly higher prevalence of CAC (62.5% vs. 0%, *P* < 0.001) (Supplementary Table S1).

Table 1 provides a summary of the demographic, dialysis-related, and biochemical characteristics for the entire cohort and its two subgroups. The CAC-positive and CAC-negative groups showed no significant differences in age, sex, smoking history, blood pressure, volume management indicators (UFR and %IDWG), phosphate binder usage,most biochemical markers, or primary kidney disease distribution (all *P* > 0.05). However, the CAC-positive group exhibited a significantly longer dialysis vintage, lower Kt/V, increased left ventricular mass index (LVMI), and elevated serum phosphate, serum calcium and ALP levels (all *P* < 0.05).

**Table 1:** Baseline characteristics of the overall study population and comparisons between patients with and without coronary artery calcification. .

Variable	All (*n* = 88)	CAC- negative (*N* = 33, 37.5%)	CAC- positive (*N* = 55, 62.5%)	*p*
Demographic data				
Age, years	69.00 [62.75, 78.00]	69.00 [62.00, 78.00]	69.00 [63.00, 77.5]	0.641
Male sex, *n* (%)	54 (61.36%)	22 (66.67%)	32 (58.18%)	0.572
Smoking history, *n* (%)				0.114
Never smoker	59 (67.05%)	26 (78.8%)	33 (60.00%)	
Former smoker	29 (32.95%)	7 (21.21%)	22 (40.00%)	
BMI, kg/m²	24.65 [23.15, 27.10]	24.49 [22.31, 25.95]	24.68 [23.36, 27.51]	0.231
Dialysis-related Parameters				
Dialysis vintage, months	26.24 [20.32, 39.61]	22.24 [18.08, 25.76]	32.00 [23.86, 50.83]	< 0.001
Kt/V	1.47 (0.11)	1.51 (0.10)	1.44 (0.10)	0.003
SBP, mm Hg	144.53 (19.31)	142.18 (22.39)	145.95 (17.28)	0.216
DBP, mm Hg	78.75 (12.93)	75.88 (12.66)	80.47 (12.89)	0.107
MAP, mm Hg	99.99 (13.98)	96.50 (15.54)	102.08 (12.65)	0.070
LVMI, g/m²	106.69 (21.54)	87.82 (11.95)	118.01 (17.75)	< 0.001
Biochemical data				
Serum phosphate, mmol/L	2.30 [1.90, 2.64]	2.28 [1.84, 2.43]	2.36 [1.95, 2.93]	0.033
Serum calcium, mmol/L	2.34 (0.42)	2.15 (0.39)	2.46 (0.40)	< 0.001
Blood platelet, × 10⁹/L	189.50 [156.75, 243.00]	183.00 [148.00, 226.00]	192.00 [163.50, 247.50]	0.219
Albumin, g/L	38.34 (2.88)	38.26 (2.45)	38.39 (3.13)	0.833
Haemoglobin, g/L	108.13 (23.79)	108.55 (24.92)	107.88 (23.32)	0.900
ALP, U/L	71.15 [68.51, 81.00]	69.00 [67.50, 71.20]	77.00 [70.00, 86.30]	< 0.001
Uric acid, µmol/L	356.10 [284.08, 411.78]	359.10 [307.90, 417.10]	353.60 [266.05, 409.50]	0.379
IDWG, %	3.26 (0.82)	3.26 (0.89)	3.25 (0.71)	0.93
UFR, mL/(h·kg)	8.18 (2.00)	8.17 (2.24)	8.19 (1.56)	0.97
Calcimimetics, *n* (%)	39 (44.3)	20 (60.6)	19 (42.2)	0.109
Phosphate binders, *n* (%)				0.89
Calcium-based binders (Calcium carbonate/acetate), *n* (%)	16 (18.18)	6 (18.18)	10 (18.18)	
Sevelamer, *n* (%)	31 (35.23)	10 (30.30)	21 (38.18)	
Lanthanum carbonate, *n* (%)	22 (25.00)	9 (27.27)	13 (23.64)	
Ferric citrate, *n* (%)	19 (21.59)	8 (24.24)	11 (20.00)	
SDC1, pg/mL	47.86 [29.36, 73.79]	25.24 [22.05, 33.46]	71.73 [45.80, 79.30]	< 0.001

Data are expressed as median [interquartile range], mean (standard deviation), or *n* (%), depending on suitability. CAC stands for coronary artery calcification; CKD refers to chronic kidney disease; Kt/V is the urea clearance index; BMI denotes body mass index; SBP is systolic blood pressure; DBP means diastolic blood pressure; MAP represents mean arterial pressure; LVMI is the left ventricular mass index; ALP stands for alkaline phosphatase. SDC1, syndecan-1. *P* values were calculated using the Mann–Whitney U test for continuous variables and the chi-square test for categorical variables.

### 3.2 Distribution and exploratory ROC analysis of SDC1

**Figure 2:**
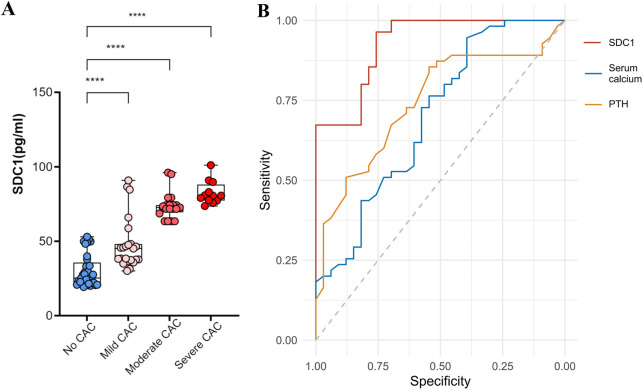
Serum SDC1 levels across CAC severity and its exploratory ROC analysis. **(A)** Box plot comparing serum SDC1 levels among patients with no, mild, moderate, and severe coronary artery calcification (CAC). SDC1 levels increased stepwise with higher CAC severity (****P < 0.0001). **(B)** Exploratory receiver operating characteristic (ROC) curves of serum SDC1, PTH, and serum calcium for identifying the presence of CAC. Serum SDC1 demonstrated a strong discriminative ability with an AUC of 0.920. Internal validation using 1,000 bootstrap iterations confirmed a 95% Confidence Interval for the AUC of 0.867–0.974 within this cohort.

As shown in Figure 2A, serum SDC1 levels increased progressively and significantly across the severity strata of CAC (from No CAC to Severe CAC, *P* < 0.0001).Exploratory receiver operating characteristic (ROC) curve analysis was employed to evaluate the discriminative ability of serum SDC1 in identifying the presence of CAC (Figure 2B). The findings demonstrated that SDC1 possessed strong discriminative ability, with an initial area under the curve (AUC) of 0.920 (*P* < 0.001), significantly outperforming traditional markers of mineral and bone disorders, including PTH (AUC = 0.744, 95% CI 0.640–0.849) and serum calcium (AUC = 0.698, 95% CI 0.582–0.815).To rigorously address potential model overfitting and validate the stability of the SDC1 diagnostic model, internal validation was performed using the bootstrap resampling method with 1,000 iterations. The bootstrap analysis yielded a highly robust 95% Confidence Interval for the AUC of 0.867–0.974. Furthermore, the 1,000 iterations validated an optimal threshold median of 34.2 pg/mL, which excellently aligned with the original maximum Youden's index-derived cut-off of 33.5 pg/mL (sensitivity 89.7%, specificity 85.2%). Importantly, the present ROC and cut-off analyses should be interpreted as strictly exploratory. Because the 33.5 pg/mL threshold was derived and internally validated within the same cohort, it inherently limits generalizability. Therefore, this cut-off should not be prematurely adopted as a clinical risk-stratification tool until it undergoes rigorous prospective validation in independent external cohorts.

### 3.3 Independent association between SDC1 and CAC severity

To rigorously evaluate the independent predictive value of SDC1 for CAC severity while preventing potential model overfitting associated with automated selection methods in a small cohort, a clinically prespecified sequential adjustment strategy was employed using multivariable ordinal logistic regression. Three progressive models were constructed: Model 1 was adjusted for demographic characteristics (age and sex); Model 2 was additionally adjusted for clinical history variables (dialysis vintage and diabetic nephropathy); Model 3 (the comprehensive model) further incorporated core markers of chronic kidney disease-mineral and bone disorder (CKD-MBD) (serum phosphate, serum calcium, and PTH). Collinearity diagnostics confirmed the absence of multicollinearity among these variables (all VIFs < 2.0, detailed in Supplementary Table S4).

**Figure 3:**
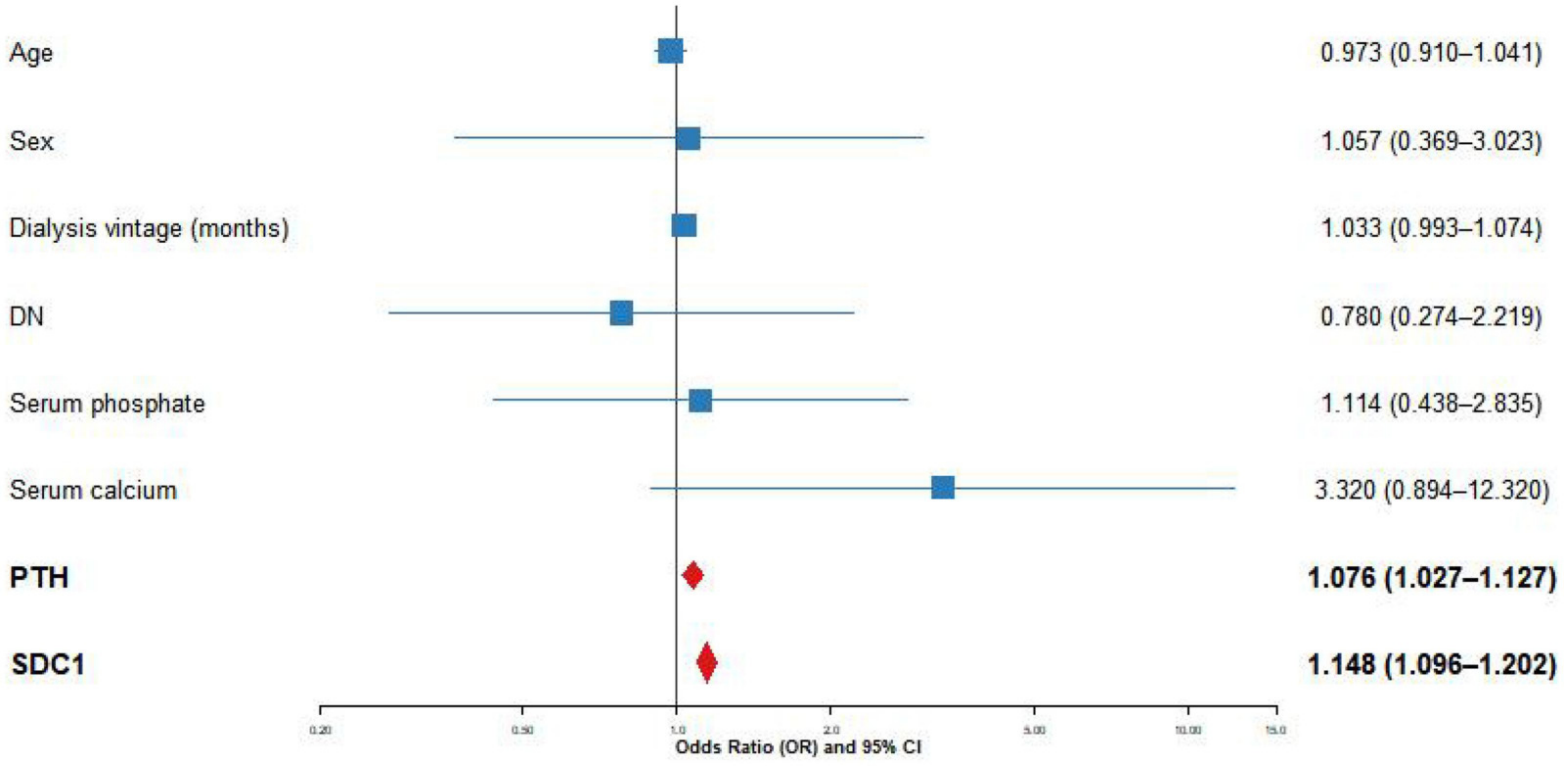
Forest plot of the comprehensive multivariable ordinal logistic regression model for CAC severity.

**Figure 4:**
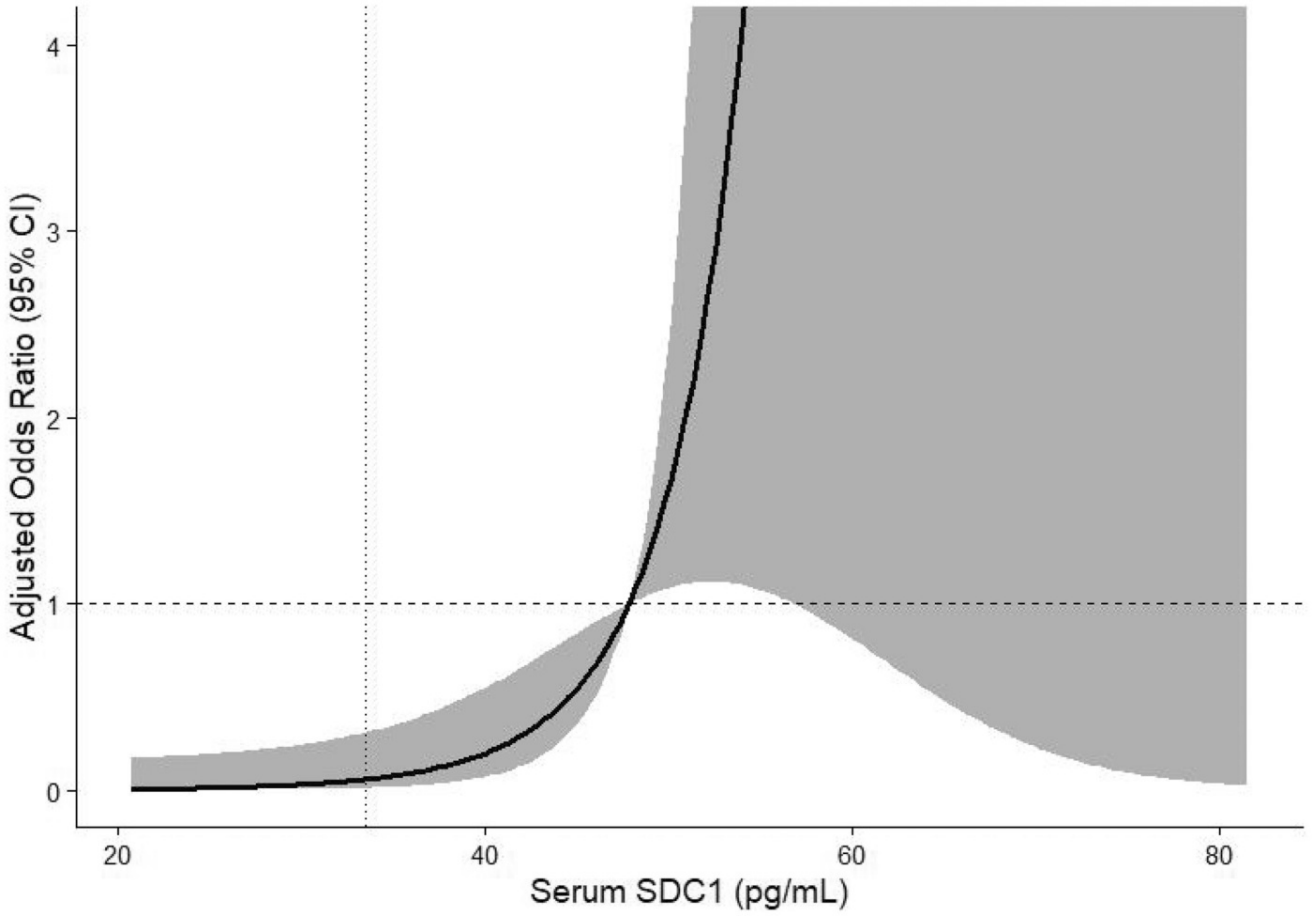
Restricted cubic spline (RCS) analysis of the association between serum SDC1 levels and the risk of coronary artery calcification. The solid line represents the adjusted odds ratio for CAC risk across continuous serum SDC1 levels, and the shaded area indicates the 95% confidence interval. A steep escalation in risk is observed near the internally validated cut-off value.

As detailed in Supplementary Table S2, elevated serum SDC1 consistently demonstrated a powerful and independent association with aggravated CAC severity across all adjustment steps. In the comprehensive model (Model 3), SDC1 remained a significant independent risk factor (Adjusted OR = 1.148, 95% CI 1.096–1.202, *P* < 0.001). Interestingly, elevated PTH was also identified as an independent predictor in this comprehensive model (Adjusted OR = 1.076, 95% CI 1.027–1.127, *P* = 0.002). This independent predictive value is further visualized in the updated Forest Plot (Figure 3). Furthermore, restricted cubic spline (RCS) analysis (Figure 4) revealed a continuous, non-linear threshold effect between serum SDC1 levels and the adjusted risk of CAC, visually corroborating the steep escalation of risk near the internally validated cut-off value.

Visualization of the independent predictors for CAC severity. To prevent overfitting, the comprehensive model (Model 3) was constructed using a clinically prespecified sequential adjustment strategy. Elevated serum SDC1 remained a powerful independent risk factor (Adjusted OR = 1.148, 95% CI 1.096–1.202, *P* < 0.001) after comprehensive adjustment for age, sex, dialysis vintage, diabetic nephropathy (DN), serum phosphate, serum calcium, and PTH.

### 3.4 Descriptive clinical characteristics stratified by the exploratory SDC1 threshold

To descriptively examine the clinical phenotypes and IDH profiles associated with different serum SDC1 levels, patients were dichotomized based on the exploratory threshold of 33.5 pg/mL into a higher SDC1 group (SDC1 ≥ 33.5 pg/mL) and a lower SDC1 group (SDC1 < 33.5 pg/m).This threshold-based stratification was applied solely to descriptively compare clinical characteristics and IDH profiles across different SDC1 levels.

Comparative analyses between these two groups are detailed in Table 2. While age and sex distributions were analogous between the groups, the higher SDC1 cohort was characterized by a longer median dialysis vintage and a significantly higher prevalence of CAC (86.9% vs. 7.4%, *P* < 0.001). Regarding the IDH profile, the higher SDC1 group experienced a higher frequency of IDH episodes over the preceding 3 months (*P* < 0.001). Concurrently, a greater proportion of patients in the higher SDC1 group had documented requirements for long-term preventive measures, including dry weight reduction, adjustment of antihypertensive medications, and cool-temperature dialysis protocols (all *P* < 0.001). These cross-sectional associations suggest a potential link between elevated SDC1, vascular calcification, and hemodynamic instability, providing a hypothesis-generating basis that requires prospective confirmation in future studies.

**Table 2:** Comparison of baseline characteristics and IDH profiles stratified by Serum SDC1 level.

Variable	higher SDC1 group (SDC1 ≥ 33.5 pg/mL) (*n* = 61)	Lower SDC1 group (SDC1 < 33.5 pg/mL) (*n* = 27)	*p*
Demographic data			
Age, years	69.00 [62.00, 77.00]	69.00 [63.00, 78.00]	0.903
Male sex, *n* (%)	36 (59.02%)	18 (66.67%)	0.658
Dialysis vintage, months	32.00 [23.28, 45.84]	22.24 [18.40, 26.36]	< 0.001
Primary Outcomes			
Coronary artery calcification, *n* (%)	53 (86.89%)	2 (7.41%)	< 0.001
SDC1, pg/mL	68.64 [46.65, 77.48]	24.27 [21.75, 28.01]	< 0.001
IDH Profile (Past 3 Months)			
IDH episodes, median [IQR]	1 [0, 2]	0 [0, 0]	< 0.001
Acute interventions during episode [*n* (%)]			
Ultrafiltration adjustment + postural change only	17 (27.87%)	0 (0.00%)	
Saline infusion (≤ 200 mL)	9 (14.75%)	0 (0.00%)	
Hypertonic solution infusion or session termination	8 (13.11%)	0 (0.00%)	
Long-term Preventive Measures [*n* (%)]			
Dry weight reduction	16 (27.12%)	0 (0.00%)	< 0.001
Adjustment of antihypertensive medications	8 (13.56%)	0 (0.00%)	< 0.001
Cool-temperature dialysis (≤ 36 °C)	8 (13.56%)	0 (0.00%)	< 0.001

Data are expressed as median [interquartile range] or *n* (%), as applicable. The SDC1 cut-off value (33.5 pg/mL) for identifying the presence of coronary artery calcification was determined using ROC curve analysis. Intradialytic hypotension (IDH) was defined as a symptomatic systolic blood pressure (SBP) drop ≥ 20 mmHg or a nadir SBP < 90 mmHg necessitating nursing intervention. *P* values were calculated using the Mann–Whitney U test for continuous variables and the Chi-square or Fisher's exact test for categorical variables. SDC1, syndecan-1.

## 4 Discussion

In this study of elderly MHD patients, we observed a high prevalence of coronary artery calcification (62.5% with a Coronary Artery Calcium Score > 0). Notably, serum SDC1, a marker of endothelial glycocalyx damage, was independently associated with both the severity of CAC and the occurrence of IDH. This connection suggests a potential shared biological pathway between structural vascular lesions and functional hemodynamic changes, providing exploratory insights into cardiovascular risk in this population (14, 15).

The significant correlation (*r* = 0.75) between SDC1 and CACS, validated as an independent biomarker, supports the advancing insights into the mechanisms of vascular calcification (16). Mineral bone disorder linked to chronic kidney disease (CKD-MBD) has traditionally been viewed as the main cause of vascular calcification in CKD patients (17, 18). However, emerging evidence suggests that endothelial dysfunction may serve as an early trigger in this process. The endothelial glycocalyx, a protective gel-like layer lining the vascular lumen, serves as the primary barrier against harmful factors (19). In addition to uremic toxins, inflammatory cytokines, and oxidative stress, the non-physiological shear stress caused by hemodialysis procedures and the bioincompatibility of dialysis membranes may further exacerbate endothelial glycocalyx damage and SDC1 shedding in MHD patients—this is a unique pathological feature of this population compared with non-dialysis CKD patients (20). Crucially, the degradation of this barrier increases vascular permeability. This aligns perfectly with our findings in the comprehensive ordinal regression model, where elevated SDC1 emerged as a significant independent variable associated with CAC severity. The compromised glycocalyx structurally facilitates the subendothelial infiltration of circulating lipids, thereby accelerating chronic inflammation and the subsequent osteogenic transformation of vascular smooth muscle cells. Furthermore, the exploratory ROC analyses demonstrated that SDC1 possessed strong discriminative ability in identifying early vascular vulnerability. This study reveals a notable association between SDC1 and IDH episodes. IDH is a prevalent acute complication of dialysis, strongly associated with adverse patient outcomes (21, 22). Historically, IDH risk has been mainly linked to cardiac function, volume status, and autonomic regulation (23). Our findings present new evidence indicating that systemic endothelial damage may significantly contribute to the underlying mechanisms of IDH. A plausible integrative explanation is that uremia-induced damage to the pan-vascular endothelial glycocalyx may result in two distinct clinical outcomes through separate pathophysiological timelines. Chronically, the compromised endothelium facilitates lipid infiltration, chronic inflammation, and osteogenic transformation of vascular smooth muscle cells, ultimately leading to vascular calcification (24). Acutely, the loss of glycocalyx is closely linked to impaired endothelium-dependent vasomotor regulation, diminishing the vasculature's capacity to swiftly adapt to hemodynamic changes induced by ultrafiltration, which may contribute to IDH (25). The independent association of the same biomarker (SDC1) with both outcomes strongly supports this hypothesis.

For patients exhibiting elevated SDC1 levels, clinical management should encompass a dual approach: firstly, addressing long-term risk by enhancing primary cardiovascular prevention strategies; and secondly, focusing on acute clinical events through the implementation of individualized dialysis prescription strategies. In the context of nursing practice, this necessitates improved real-time monitoring of blood pressure and symptoms during dialysis for high-risk patients, alongside the proactive development and rigorous execution of graded IDH emergency protocols, such as adjustments in ultrafiltration and pre-planned fluid supplementation strategies. Additionally, it involves the meticulous application of preventive measures, including precise dry weight assessment, coordinated management of antihypertensive medications on dialysis days, and the effective use of cool-temperature dialysis. Moreover, SDC1 serves as a potential objective biological marker for assessing the efficacy of novel interventions aimed at enhancing endothelial function, such as specific anti-inflammatory or antioxidant therapies.

Limitations and Future Directions: This study has several constraints. First, its cross-sectional design precludes establishing causality between SDC1, CAC, and IDH. Second, the healthy controls were matched only by age, and the retrospective nature of clinical data extraction meant certain confounders, such as statin use and precise calcium load, could not be fully accounted for. Third, we utilized total serum ALP rather than bone-specific isoforms, which may introduce non-skeletal noise. Fourth, although our sample size was statistically justified, the single-center scope necessitates further validation in larger, multicenter cohorts. Crucially, as the SDC1 threshold was derived and internally validated within the same cohort, it remains preliminary and requires rigorous external validation before clinical application. Future longitudinal research is essential to determine whether SDC1 can forecast hard clinical endpoints like MACE and mortality, while mechanistic studies are needed to further elucidate the biological pathways involved.

## 5 Conclusions

This exploratory study demonstrates that elevated serum SDC1 is independently associated with the severity of coronary artery calcification and the frequency of intradialytic hypotension in elderly MHD patients. These findings suggest that endothelial glycocalyx degradation may represent a potential bridging link between chronic structural vascular lesions and acute functional hemodynamic instability. From a clinical perspective, SDC1 profiling may hold potential for identifying patients with high vascular vulnerability, supporting a transition from reactive to more proactive, individualized nursing care. If validated in future prospective studies, integrating this biomarker into clinical assessment could facilitate the implementation of anticipatory blood pressure monitoring and tailored dialytic prescriptions. Such a nursing-led, multifaceted approach would be essential for optimizing hemodynamic stability, patient safety, and long-term outcomes in the elderly hemodialysis population.

## Data Availability

The original contributions presented in the study are included in the article/Supplementary Material, further inquiries can be directed to the corresponding authors.
