# Supplementary material for: Serum syndecan-1 correlates with coronary artery calcification severity and intradialytic hypotension in elderly hemodialysis patients
Source: Front Cardiovasc Med. 2026 Jun 11;13:1842977. doi: 10.3389/fcvm.2026.1842977 (PMC13293849; doi:10.3389/fcvm.2026.1842977)

Supplementary Table S1. Baseline Characteristics of Elderly Maintenance Hemodialysis Patients and Age-Matched Healthy Controls.

| **Variable** | **Patients (n=88)** | **Healthy Controls**  **(n=30)** | ***p*-value** |
| --- | --- | --- | --- |
| Age, years | 69.00 [62.75, 78.00] | 67.50 [65.00, 70.75] | 0.202 |
| Male sex, n (%) | 54(61.36%) | 16(53.33%) | 0.440 |
| Dialysis vintage, months | 26.24 [20.32, 39.61] |  |  |
| **Primary cause of CKD, n (%)** |  |  |  |
| ANCA-associated vasculitis | 1 (1.14%) |  |  |
| Polycystic kidney disease | 2 (2.27%) |  |  |
| Hypertensive nephropathy | 10 (11.36%) |  |  |
| Obstructive nephropathy | 7 (7.95%) |  |  |
| Chronic Nephritis | 17 (19.32%) |  |  |
| Membranous nephropathy | 1 (1.14%) |  |  |
| Nephrotic syndrome | 6 (6.82%) |  |  |
| Hyperuricemia nephropathy | 2 (2.27%) |  |  |
| Diabetic nephropathy | 42 (47.73%) |  |  |
| Coronary artery calcification, n (%) | **55** (**62.5%**) | 0 | < 0.001 |
| SDC1, pg/mL | 47.86 [29.36, 73.79] | 15.28 [13.44, 17.72] | < 0.001 |

Data are presented as median [interquartile range] or n (%), as appropriate. The 30 healthy controls were retrospectively screened to confirm a zero coronary artery calcium score (CACS = 0) to establish a strict physiological baseline. SDC1, syndecan-1.

Supplementary Table S2. Sequential multivariable ordinal logistic regression analysis evaluating the independent association of serum SDC1 with CAC severity.

| **Variable** | **Model 1 OR**  **(95% CI); *P*-value** | **Model 2 OR**  **(95% CI); *P*-value** | **Model 3 OR**  **(95% CI); *P*-value** |
| --- | --- | --- | --- |
| SDC1 (per 1 pg/mL increase) | 1.146(1.104 **–**1.189) *P*<0.001 | 1.136(1.092 **–** 1.182)  *P*<0.001 | 1.148(1.096 **–** 1.202)  *P*<0.001 |
| **Covariates Adjusted:** |  |  |  |
| Age and Sex | Yes | Yes | Yes |
| Dialysis vintage & Diabetic nephropathy | No | Yes | Yes |
| CKD-MBD markers (P, Ca, PTH) | No | No | Yes |

Note: CAC, coronary artery calcification; CI, confidence interval; CKD-MBD, chronic kidney disease-mineral and bone disorder; OR, odds ratio; PTH, parathyroid hormone; SDC1, syndecan-1.

Model 1: Adjusted for demographic variables (Age and Sex).

Model 2: Adjusted for Model 1 variables plus clinical history (Dialysis vintage and Diabetic nephropathy).

Model 3 (Comprehensive model): Adjusted for Model 2 variables plus core CKD-MBD parameters (Serum phosphate, Serum calcium, and PTH).

Supplementary Table S3. Baseline characteristics and intradialytic hypotension (IDH) profiles stratified by coronary artery calcification severity.

| **Variable** | **Overall, N = 88** | **No CAC, N=33**  **(37%)** | **Mild CAC, N=23**  **(26%)** | **Moderate CAC, N=20**  **(23%)** | **Severe CAC, N=12**  **(14%)** | **p-value** |
| --- | --- | --- | --- | --- | --- | --- |
| Age, years | 69.00 [62.75, 78.00] | 69.00 [62.00, 78.00] | 69.00 [65.00, 73.00] | 68.00 [62.00, 79.00] | 70.50 [61.75, 78.75] | 0.965 |
| Male sex, n (%) | 54 (61.36%) | 22 (66.67%) | 10 (43.48%) | 13 (65.00%) | 9 (75.00%) | 0.257 |
| Dialysis vintage, months | 26.24 [20.32, 39.61] | 22.24 [18.08, 25.76] | 28.28 [22.70, 34.00] | 39.24 [22.29, 46.80] | 59.43 [39.53, 67.91] | <0.001 |
| BMI, kg/m² | 24.65 [23.15, 27.10] | 24.49 [22.31, 25.95] | 23.39 [22.68, 25.71] | 25.44 [23.80, 27.33] | 27.47 [24.61, 29.26] | 0.048 |
| SBP, mm Hg | 144.53 (19.31) | 142.18 (22.39) | 145.52 (18.60) | 143.55 (18.07) | 150.75 (13.18) | 0.343 |
| DBP, mm Hg | 78.75 (12.93) | 75.88 (12.66) | 76.96 (14.08) | 78.60 (9.93) | 90.33 (10.50) | 0.007 |
| MAP, mm Hg | 99.99 (13.98) | 96.50 (15.54) | 99.58 (13.34) | 100.03 (11.20) | 110.27 (10.90) | 0.033 |
| Haemoglobin, g/L | 108.13 (23.79) | 108.55 (24.92) | 101.43 (22.47) | 111.48 (22.38) | 114.24 (25.30) | 0.394 |
| Serum calcium, mmol/L | 2.34 (0.42) | 2.15 (0.39) | 2.38 (0.40) | 2.47 (0.40) | 2.58 (0.37) | 0.003 |
| Serum phosphate, mmol/L | 2.30 [1.90, 2.64] | 2.28 [1.84, 2.43] | 2.12 [1.88, 2.33] | 2.73 [2.19, 3.07] | 2.76 [2.21, 3.05] | 0.002 |
| ALP, U/L | 71.15 [68.51, 81.00] | 69.00 [67.50, 71.20] | 71.10 [70.00, 79.50] | 81.40 [70.00, 90.25] | 76.80 [72.00, 88.20] | <0.001 |
| Uric acid, µmol/L | 356.10 [284.08, 411.78] | 359.10 [307.90, 417.10] | 370.30 [305.15, 403.50] | 328.60 [261.25, 408.25] | 337.50 [266.00, 433.25] | 0.587 |
| IL6, pg/mL | 29.81 [25.82, 33.93] | 28.51 [23.84, 30.59] | 30.82 [29.02, 33.32] | 30.28 [26.96, 36.01] | 31.86 [26.50, 37.15] | 0.026 |
| Homocysteine, pg/mL | 21.40 [18.98, 24.43] | 21.60 [18.90, 25.40] | 20.10 [17.95, 22.70] | 21.30 [19.20, 23.75] | 22.50 [21.33, 24.09] | 0.440 |
| Total Cholesterol, mmol/L | 4.49 [3.93, 5.57] | 4.12 [3.22, 4.63] | 4.47 [4.00, 5.06] | 5.19 [4.30, 5.99] | 6.65 [5.52, 7.55] | <0.001 |
| Triglyceride, mmol/L | 1.62 [1.24, 2.23] | 1.62 [1.23, 2.55] | 1.60 [1.24, 2.00] | 1.78 [1.31, 2.52] | 1.40 [1.29, 1.72] | 0.643 |
| HDL-C, mmol/L | 1.17 [1.01, 1.45] | 1.10 [0.93, 1.39] | 1.27 [1.03, 1.60] | 1.29 [1.15, 1.79] | 1.08 [0.97, 1.27] | 0.041 |
| LDL-C, mmol/L | 2.23[1.42, 3.27] | 2.13[1.29, 2.79] | 2.11[1.42, 2.60] | 2.85[1.77, 4.50] | 2.38[1.49, 3.74] | 0.017 |
| LpPLA2, ng/mL | 262.16 (21.79) | 259.52 (21.53) | 258.39 (21.83) | 260.99 (20.40) | 278.64 (19.62) | 0.041 |
| PTH, pg/mL | 83.20 [78.00, 90.75] | 78.40 [73.20, 83.40] | 89.00 [83.20, 94.50] | 82.60 [79.47, 95.45] | 86.54 [82.40, 88.85] | 0.001 |
| SDC1, pg/mL | 47.86 [29.36, 73.79] | 25.24 [22.05, 33.46] | 45.15 [37.56, 47.86] | 72.67 [70.96, 74.25] | 80.66 [77.43, 84.74] | <0.001 |
| IDH Episodes & Management (Past 3 Months) |  |  |  |  |  | <0.001 |
| Frequency of IDH episodes (median [IQR]) | 0 [0-2] | 0 [0-1] | 1 [0-2] | 1 [0-4.25] | 1.5 [0.75-5.5] |  |
| Acute interventions during episode [n (%)] |  |  |  |  |  | 0.031 |
| Ultrafiltration adjustment + postural change only | 17 (19.32%) | 4 (12.12%) | 5 (21.74%) | 4 (20.00%) | 4 (33.33%) |  |
| Saline infusion (≤200 mL) | 9 (10.23%) | 1 (3.03%) | 2 (8.70%) | 4 (20.00%) | 2 (16.67%) |  |
| Hypertonic solution infusion or session termination | 8 (9.09%) | 1 (3.03%) | 3 (13.04%) | 1 (5.00%) | 3 (25.00%) |  |
| Long-term Preventive Measures [n (%)] |  |  |  |  |  | 0.029 |
| Dry weight reduction | 16 (18.60%) | 2 (6.06%) | 5 (23.81%) | 5 (25.00%) | 4 (33.33%) |  |
| Adjustment of antihypertensive medications | 8 (9.30%) | 3 (9.09%) | 1 (4.76%) | 2 (10.00%) | 2 (16.67%) |  |
| Cool-temperature dialysis (≤36°C) | 8 (9.30%) | 1 (3.03%) | 2 (9.52%) | 2 (10.00%) | 3 (25.00%) |  |

Data are expressed as median [interquartile range], mean (standard deviation), or n (%), as appropriate. CAC severity was categorized based on the coronary artery calcium score. Intradialytic hypotension (IDH) was defined as a symptomatic systolic blood pressure (SBP) drop ≥ 20 mmHg or a nadir SBP < 90 mmHg necessitating nursing intervention. Abbreviations: CAC, coronary artery calcification; HDL-C, high-density lipoprotein cholesterol; IDH, intradialytic hypotension; LDL-C, low-density lipoprotein cholesterol; SDC1, syndecan-1.

Supplementary Table S4. Collinearity diagnostics for the comprehensive multivariable ordinal logistic regression model.

| **Variable** | **VIF Value** | **Tolerance** |
| --- | --- | --- |
| Age | 1.10 | 0.91 |
| Sex | 1.11 | 0.90 |
| Dialysis vintage (months) | 1.75 | 0.57 |
| DN | 1.08 | 0.93 |
| Serum phosphate | 1.36 | 0.74 |
| Serum calcium | 1.17 | 0.86 |
| PTH | 1.11 | 0.90 |
| SDC1 | 1.96 | 0.51 |

Collinearity among the covariates in the comprehensive model (Model 3) was strictly ruled out, with all Variance Inflation Factor (VIF) values being exceptionally low (< 2.0). PTH, parathyroid hormone; SDC1, syndecan-1; VIF, variance inflation factor.

Supplementary Figure S1. Standardized clinical protocol for intradialytic hypotension (IDH) monitoring and management.


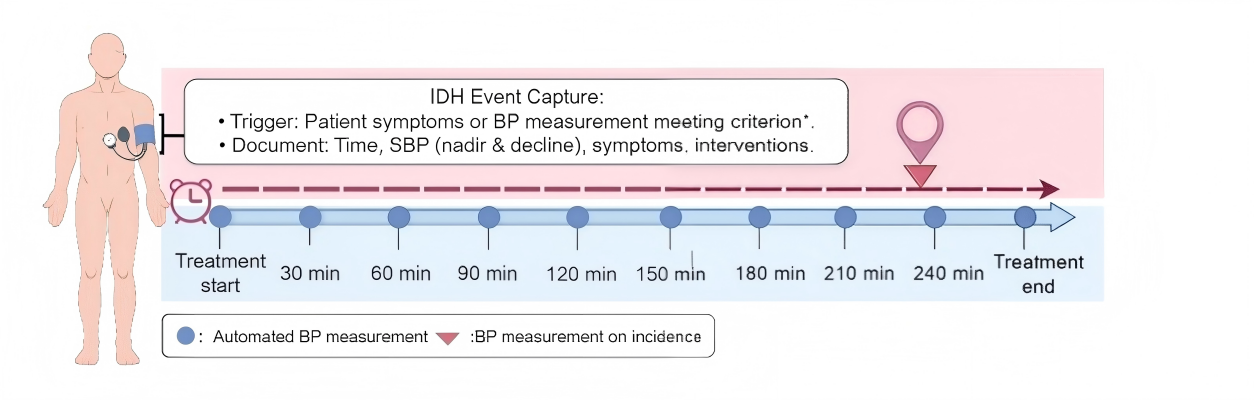
Blood pressure was routinely monitored at 30-minute intervals throughout each hemodialysis session using automated oscillators. An IDH event was defined and documented by trained nursing staff when a patient experien ced a symptomatic systolic blood pressure (SBP) drop ≥ 20 mmHg or a nadir SBP < 90 mmHg necessitating nursing intervention. All event details and therapeutic interventions were recorded in real-time within a standardized electronic medical record system to ensure data accuracy and minimize retrospective recall bias.

Supplementary Figure S2. Correlation heatmap between coronary artery calcium score (CACS) and laboratory variables.

Colors represent Spearman's rank correlation coefficients (rs) for all variables, with purple indicating positive correlations and blue indicating negative correlations. Darker shades denote stronger correlation magnitudes. Correlations with P < 0.05 were considered statistically significant; for clinical interpretability, we focused on correlations with |rs| ≥ 0.25 as meaningful. CACS showed the strongest positive correlation with serum SDC1 (rs = 0.75, P < 0.001), followed by dialysis vintage (rs = 0.66, P < 0.001).

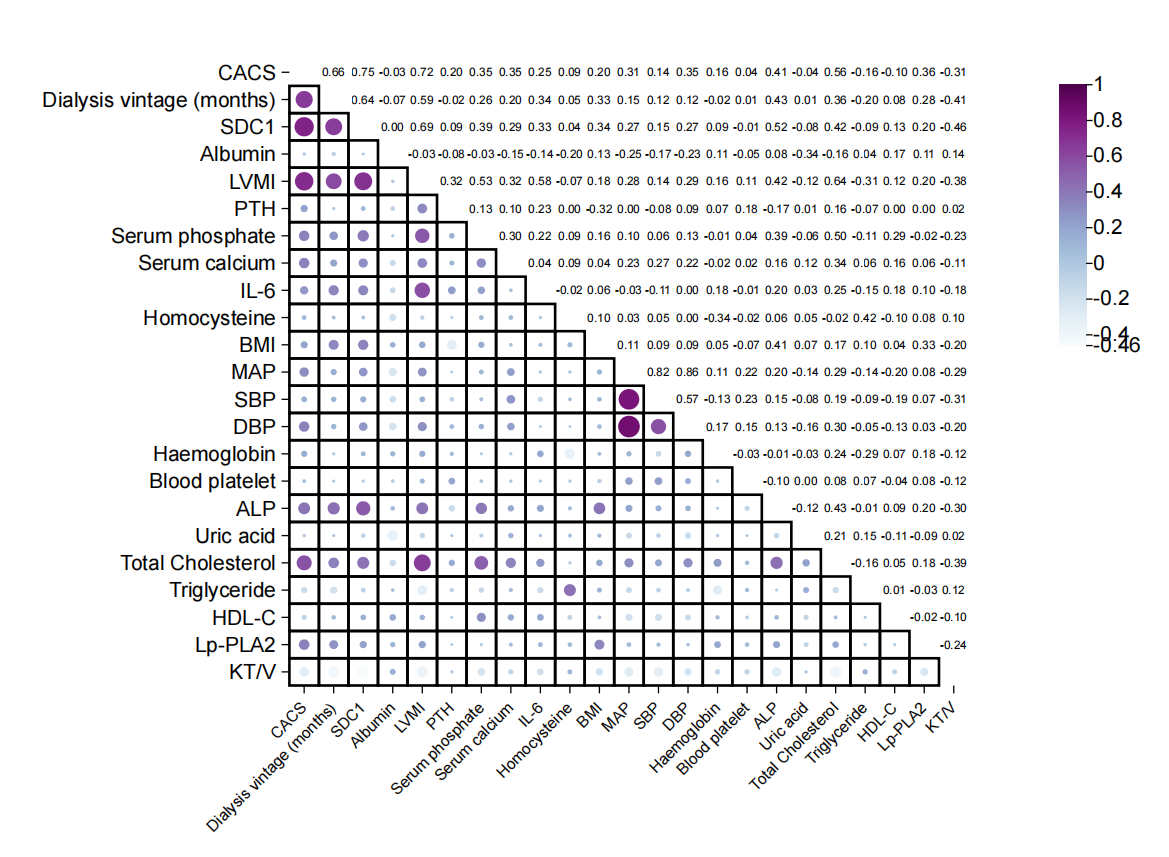

Supplement: Supplementary file 1 [file Table1.docx]
